# Highest Frequencies of Interleukin-22-Producing T Helper Cells in Alcoholic Hepatitis Patients with a Favourable Short-Term Course

**DOI:** 10.1371/journal.pone.0055101

**Published:** 2013-01-25

**Authors:** Sidsel Støy, Thomas Damgaard Sandahl, Anders Kirch Dige, Jørgen Agnholt, Tue Kruse Rasmussen, Henning Grønbæk, Bent Deleuran, Hendrik Vilstrup

**Affiliations:** 1 Department of Medicine V (Hepatology and Gastroenterology), Aarhus University Hospital, Aarhus, Denmark; 2 Institute of Biomedicine, Aarhus University, Aarhus, Denmark; French National Centre for Scientific Research, France

## Abstract

**Background:**

Alcoholic hepatitis (AH) has a severe prognosis due to hepatic inflammatory injury. The cytokine interleukin-22 (IL-22) is reported to exert anti-apoptotic and proliferative effects, but IL-22 has not been studied during the course of AH. IL-22 is mainly produced by CD4^+^ (helper) T cells, including Th17 cells. In addition, Th17 cells produce the proinflammatory cytokine IL-17A, which has been implicated in AH.

**Aims:**

We aimed to study the levels of circulating IL-22- and IL-17A-producing T helper cells and plasma cytokines in patients with AH and to examine the observations in relation to the short-term disease course.

**Methods:**

We collected blood samples from 21 consecutive patients with severe AH on days 0, 14 and 30 after diagnosis, and included 10 stable alcoholic cirrhosis patients and 10 healthy subjects as controls. Analyses were performed using flow cytometry and ELISA.

**Results:**

We found higher frequencies of IL-22-producing T helper cells in AH patients (median 1.7%) than in cirrhosis patients (1.0%, *p* = 0.03) and healthy controls (1.0%, *p* = 0.01), and a 1.5-fold increase in the plasma concentration of IL-17A in AH compared with healthy controls (*p*<0.01). Those patients who markedly improved their Glasgow Alcoholic Hepatitis Score demonstrated a 2-fold higher frequency of IL-22-producing T helper cells at baseline and during follow-up than patients whose condition deteriorated (*p* = 0.04).

**Conclusions:**

The frequency of IL-22-producing T helper cells was increased in AH patients and most so in those whose condition seemed to improve. T cell differentiation toward an IL-22-producing phenotype may thus be favourable in AH.

## Introduction

Alcoholic hepatitis (AH) has an increasing incidence and is the most dangerous type of alcoholic liver disease with a six-month mortality of up to 40% [1], [2]. The mortality has not been possible to reduce convincingly over time, as there has been no break-through in the understanding of the immunopathology underlying the disease that can be utilized as a basis for new therapeutic interventions.

The initial innate immune response leading to alcoholic hepatitis may be triggered by alcohol locally in the liver and via the increased intestinal translocation of LPS, which activates hepatic Kupffer cells and recruits dysfunctional neutrophils [3], [4], [5]. The background of the severe disease prognosis probably is that this response is dysregulated and results in a florid activation of the adaptive immune system in the liver causing further inflammatory injury [6], [7]. Still, few studies address the involvement of the adaptive immune system in AH; however, recent reports support a possible hepato-protective and -regenerative role of the cytokine interleukin-22 (IL-22) [8]. T helper (Th) cells are the main source of IL-22, and the subsets Th1, Th17 and Th22 are responsible for its production [9], [10], [11], [12]. IL-22 is a member of the IL-10 superfamily and signals via receptors on non-hematopoietic cells, such as hepatocytes [13], [14]. Receptor engagement induces anti-apoptosis and proliferation of target cells [8], [15], [16]. IL-22 reduces alcohol-induced liver injury in mice and increases the hepatic expression of IL-22 receptors in patients with AH [17]. To the opposite effect, a high number of cells, which produce the potent proinflammatory, neutrophil-recruiting cytokine IL-17, are reported in liver biopsies from patients with AH [18]. Because the Th17 cells produce both cytokines, this subset is of obvious interest in the study of the role and interrelationship of these possibly mutually counteractive cytokines. The differentiation of Th17 cells depends on several cytokines, including IL-21 and IL-23 [19], [20]. Taken together, these reports suggest the Th17-related cytokines as targets for immunological studies in AH, where the temporal changes in Th17-related cytokines or their relation to measures of clinical outcome have not been examined.

On this background, the present study was designed to gain new insights into the dynamics of the Th17-related cytokines IL-17A and IL-22 during the course of severe AH. We hypothesized that T helper cells producing IL-22, IL-17A and IL-21 as well as the plasma cytokine levels will be elevated in AH. We, furthermore, examined whether there may be an association between high frequencies of IL-22-producing T helper cells and disease improvement, and, conversely, between high frequencies of IL-17A-producing T helper cells and disease deterioration.

## Materials and Methods

### Ethics Statement

All of the participants provided written informed consent, and The Central Denmark Region Committee on Biomedical Research Ethics approved the study (j.no. 20100281).

### Patients

A total of 21 consecutive patients were included in the study when they were diagnosed with severe AH according to the following criteria: a history of excessive alcohol ingestion until at least three weeks before admission; acute jaundice (serum bilirubin >80 µmol/l); a modified Maddrey’s discriminant function >32; the absence of infectious foci; and the absence of liver tumours or other cancers [21]. Patients were excluded from the study if they developed clinical or microbiological evidence of infection (positive chest radiographs or routine cultures of urine, blood, sputum and ascites). Other exclusion criteria included an age below 18 years or above 75 years, gastrointestinal bleeding within the past 3 months or any prior immune-modulating therapy. The clinical outcome measure of the AH patients was a change in the Glasgow Alcoholic Hepatitis Score (GAHS). A reduction in the score of ≥2 was defined as a clinical improvement. The baseline characteristics including the measurements of disease activity were not different between the two groups of AH patients (Table 1). Baseline blood samples were collected at the time of admission before the initiation of treatment. The patients were treated with 400 mg pentoxifylline TID. To elucidate whether our results are related to the effects of the severe hepatic inflammation in AH or an effect of long-term alcohol exposure, we included patients with stable alcoholic cirrhosis as sick controls (n = 10). Additionally, age- and sex-matched healthy volunteers with no history of liver or other diseases were included as healthy controls (n = 10). All patients and controls were abstinent from alcohol for at least one week preceding the investigation.

**Table 1:** Patient baseline characteristics.

	Healthy controls		Alcoholic cirrhosis		Alcoholic hepatitis				
					*↓GAHS≥2^a^*		*↓GAHS<2^b^*		P = *
Gender: F/M	4/6		2/8		2/6		4/9		
Age (years)	53	(12.0)	51	(7.0)	52	(10.5)	55	(6.0)	0.36
Weight (kg)			67	(23.0)	79	(24.5)	75	(26.0)	0.88
Height (cm)			178	(12.0)	180	(15.5)	174	(13.0)	0.56
BMI			21.5	(5.3)	24.1	(4.4)	24.8	(5.3)	0.64
ALT (U/l)			29	(21.0)	49	(46.0)	31	(37.0)	0.19
Sodium (mmol/l)			136	(6.0)	132	(8.0)	133	(5.0)	0.64
Bilirubin (µmol/l)			31	(8.0)	347	(139.5)	411	(325.0)	0.66
Alkaline phosphatase (U/l)			156	(165.0)	204	(152.0)	203	(136.0)	0.85
Hemoglobin (mmol/l)			7.4	(1.8)	7	(0.6)	6.6	(1.5)	0.26
Creatinine (µmol/l)			60	(41.0)	80	(34.5)	70	(78.0)	0.83
INR			1.4	(0.3)	1.8	(0.4)	2	(0.6)	0.06
Albumin (g/l)			31	(6.8)	27	(11.0)	29	(4.8)	0.80
MELD			11	(7.0)	25	(4.5)	24	(16.0)	0.88
GAHS			6	(2.0)	10	(2.0)	9	(2.0)	0.87
Child Pugh Score			7	(1.0)	11	(1.5)	12	(1.0)	0.50

Baseline characteristics are presented for healthy controls, stable alcoholic cirrhosis patients and alcoholic hepatitis patients. The alcoholic hepatitis patients are divided into two groups based on whether or not they experience a decline in GAHS≥2 during the 30 days of follow-up. Values are reported as median (IQR).

ALT = Alanine Amino Transferase.

MELD = Model of End Stage Liver Disease.

GAHS = Glasgow Alcoholic Hepatitis Score.

* a vs. b.

### Flow Cytometry

#### Culture and stimulation

Flow cytometry was performed on peripheral blood mononuclear cells that were isolated by Ficoll-Hypaque centrifugation (GE Healthcare Bio-sciences, Uppsala, Sweden) and stored at −140°C until analysis. The samples from each patient were collectively analysed. For the flow cytometric analysis, the cells were thawed and washed (PBS solution containing 20% heat-inactivated pooled human serum-AB). Next, the cells (2×10^∧^6/ml) were cultured in a flat-bottomed 6-well plate (Nunc, Denmark) for 4 hours at 37°C and 5% CO_2_ in culture medium (RPMI 1640 supplemented with 100 U/ml penicillin, 100 µg/ml streptomycin and 10% heat-inactivated pooled human serum-AB). The stimulation during culture consisted of 50 µg/ml phorbol-12-myristate-13-acetate (PMA) (Sigma-Aldrich, Denmark), 1 µg/ml ionomycin (Sigma-Aldrich) and 10 µg/ml of the golgi-blocking agent brefeldin A (Sigma-Aldrich) per 2.5 ml cell suspension.

#### Staining

The cultured cells were harvested, washed twice in wash buffer (PBS with 2% bovine serum albumin (BSA) and 0.9% azide) and adjusted to a concentration of 5×10^∧^6 cells/ml. The cell suspension (100 µl) was incubated with optimized amounts of fluorescent-conjugated antibodies against surface markers (CD45RO-FITC and CD4-PerCp, BD Bioscience, San Diego, CA) for 20 minutes at room temperature in the dark. Following the surface staining, the cells were washed and treated with 1.5 ml of BD FACS lysing solution (diluted 1∶10 in deionized water according to the manufacturer’s instructions, BD Bioscience), followed by permeabilisation (FACS Permeabilising Solution 2 diluted 1∶10 in deionized water according to the manufacturer’s instructions, BD Bioscience). Next, the cells were blocked with heat-inactivated murine serum (Invitrogen, Paisley, UK) and subsequently stained with fluorescent-conjugated antibodies that were directed against intracellular cytokines (IL-17A-Alexa Flour 647, IL-21-PE; eBioscience, San Diego, CA, and IL-22-PE; R&D systems, Minneapolis, MN) along with isotype controls. The isotype controls were matched by IgG subtype, concentration, species and fluorochrome. Following 20 minutes of incubation at room temperature in the dark, the cells were washed twice and fixated (250 µl PBS with 1% formaldehyde). The samples were analysed within 24 hours of preparation using a FACS Canto II instrument (BD Bioscience).

#### Gating

Based on a forward side scatter plot, a lymphocyte gate was inserted and 100,000 events were recorded within the gate. A plot of forward scatter height versus amplitude was used to exclude events without a single cell appearance. The lymphocytes were further identified based on their expression of CD4 (T helper cell) and CD45RO (activated/memory cells). FMOs (fluorescence minus one) were used to correct for data spread effects due to compensation and isotypes were used to assess unspecific binding of antibodies. We report the frequency of cytokine positive cells within the CD4^+^CD45RO^+^ population and their median fluorescence intensity (MFI).

### ELISA

Plasma was simultaneously collected with the samples for flow cytometry and stored at −80°C until use. The plasma concentrations of IL-17A, IL-21, IL-22 and IL-23 (p19/p40) were measured with ELISA kits (eBioscience) according to a previous study [22]. The samples and standards were analysed in duplicate, and the average optical density, minus the average value of the blanks, was used to calculate the concentration of a given sample based on the standard curve. The lower detection limit was determined as the value of the average of the blanks plus 2 standard deviations, which resulted in cut-off values of 2.72 pg/ml (IL-17A), 7.36 pg/ml (IL-21), 9.04 pg/ml (IL-22) and 8.40 (Il-23) pg/ml. The values below the detection limit were assigned the cut-off value.

### Statistical Analyses

We used the Kruskal-Wallis test and the Wilcoxon rank-sum test to examine the baseline differences among the groups (AH, alcoholic cirrhosis and healthy controls). In the AH patients, an ANOVA for repeated measurements was performed for a comparison of the cell and cytokine levels over time. These levels were normally distributed after a logarithmic transformation and were included in the analyses. Histograms and Q–Q plots were used to check normality, and Levene’s test was used to test the homogeneity of variance. The results are expressed as the median and interquartile range (25–75). A two-tailed *p*-value of <0.05 was considered statistically significant.

## Results

### Distinct CD4^+^CD45RO^+^ T cells were Responsible for the Production of IL-17A, IL-21 and IL-22

**Figure 1:**
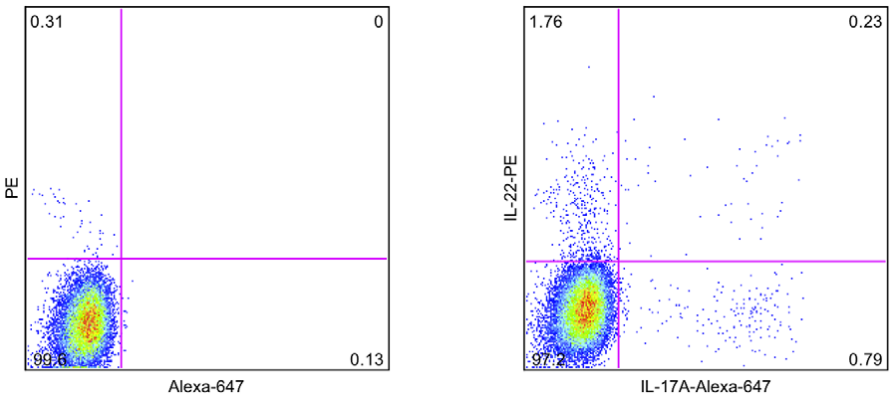
Flow plot of cytokine production. Peripheral blood mononuclear cells were stimulated with phorbol-12-myristate-13-acetate, ionomycin and brefeldin A for 4 hours followed by intracellular staining for IL-17A and IL-22. The flow plots depict the population of CD4^+^CD45RO^+^ T cells from a representative AH patient. The values in the gate represent the percentage of IL-17A^+^, IL-22^+^ and double positive cells within the population of CD4^+^CD45RO^+^ T cells (right). The gate is set based on the control staining (left).

Th17 cells are reported to co-express IL-17A, IL-21 and IL-22 [23]. Therefore, we concomitantly stained for these cytokines to determine whether Th17 cells producing all three cytokines were present in the circulation of AH patients. We observed that the majority of cytokine-producing cells were detected inside the lymphocyte gate (data not shown). The cytokine-producing cells were, however, predominantly single positive for one of the cytokines (Figure 1); therefore, we evaluated the cytokine-positive cell populations separately throughout the study.

### Elevated Frequency of IL-22-producing CD4^+^CD45RO^+^ T cells in AH

**Figure 2:**
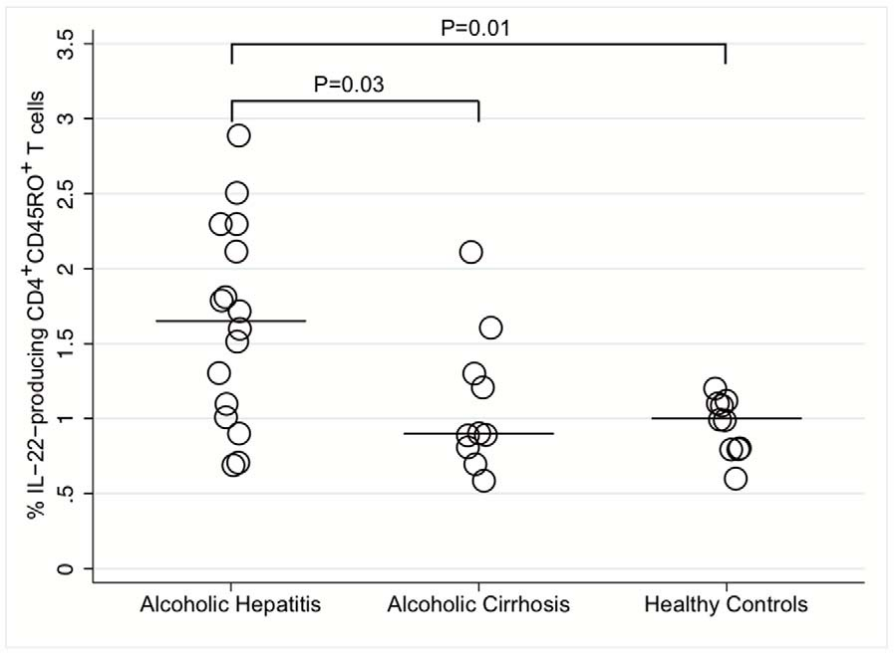
Baseline IL-22-producing T helper cells. Flow cytometric measurement of the percentages of IL-22-producing CD4^+^CD45RO^+^ T cells in AH patients on day 0, alcoholic cirrhosis patients and healthy controls in peripheral blood. The frequency of IL-22-producing CD4^+^CD45RO^+^ T cells are increased in AH patients compared with both of the controls groups. The bars represent median values.

**Figure 3:**
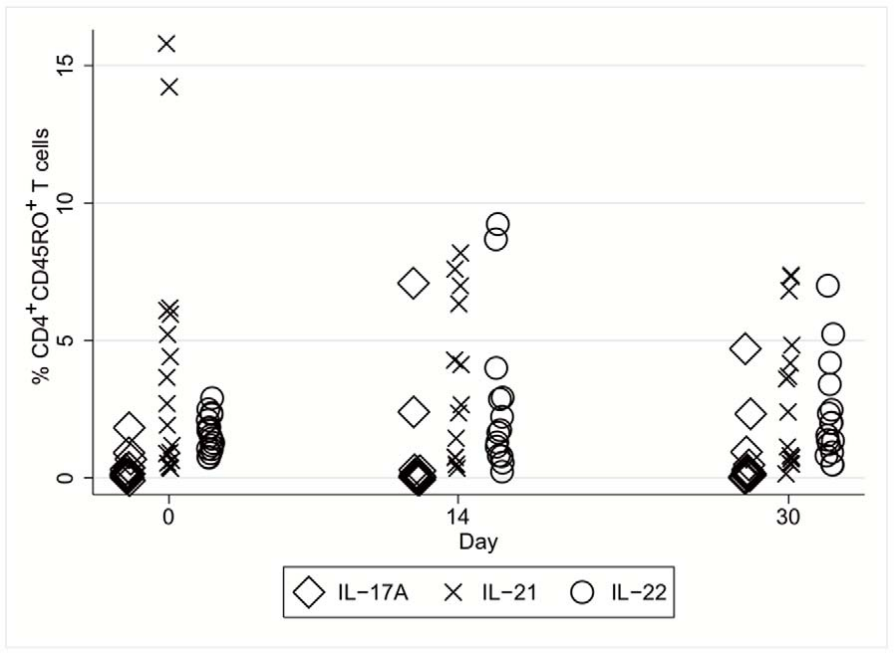
Frequency of cytokine producing T helper cells during follow-up. The percentages of CD4^+^CD45RO^+^ T cells that produced IL-17A, IL-21 or IL-22 in patients with AH on days 0, 14 and 30 after diagnosis as measured by flow cytometry. We observed no changes in the frequencies of neither of these cells during this 30-days follow-up period.

We measured the percentages of CD4^+^CD45RO^+^ T cells that produced IL-22 in the peripheral blood at baseline and detected a 1.7-fold increase in the frequency of these cells in AH patients compared with both alcoholic cirrhosis patients and healthy controls. This increase was significant (Figure 2). The average amount of IL-22 produced per CD4^+^CD45RO^+^ T cells was not different between the groups as the populations MFI did not differ (data not shown). During follow-up after 2 and 4 weeks of treatment, the frequencies of IL-22-producing CD4+CD45RO+ T cells remained unchanged (Figure 3, Table 2). Having shown an increased number of IL-22-producing CD4^+^CD45RO^+^ T cells in active AH, we progressed to examine if this was reflected in IL-22 plasma levels. However, plasma concentrations of IL-22 were not different among the groups (Table 2).

**Table 2:** Plasma cytokine concentrations.

	Healthy controls		Alcoholic cirrhosis		Alcoholic hepatitis					
Cytokine					day 0		day 14		day 30	
IL-17A	2.7	(0.0)	3.0	(1.8)	3.5*	(2.2)	3.1	(1.4)	4.0	(1.6)
IL-21	12.7	(3.6)	13.2	(26.6)	15.1	(12.9)	15.1	(12.2)	14.1	(10.6)
IL-22	27.0	(33.0)	40.1	(129.6)	36.7	(50.1)	18.0	(30.7)	32.8	(50.7)
IL-23	8.4	(1.5)	8.6	(8.3)	8.4	(0.0)	8.4	(0.0)	8.4	(0.2)

The concentrations of cytokines in plasma were measured with ELISA in healthy controls, alcoholic cirrhosis and for alcoholic hepatitis patients at day 0, 14 and 30 after diagnosis. The concentrations are reported in pg/ml. Values are presented as median (IQR).

* AH day 0 compared with healthy controls (*p*<0.01).

### High Frequency of IL-22-producing CD4^+^CD45RO^+^ T cells in Patients whose Condition Improved

**Figure 4:**
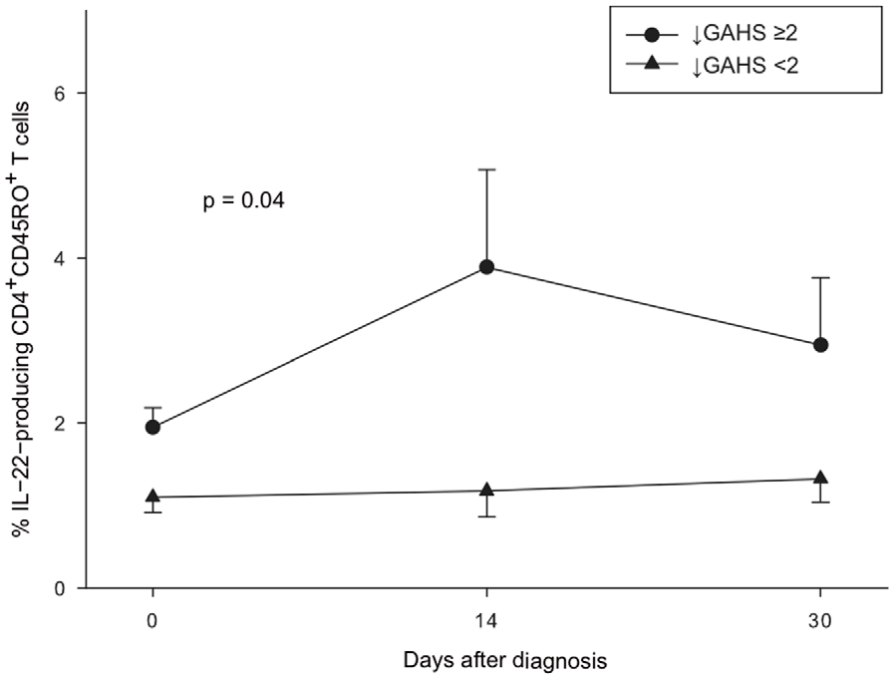
Clinical disease course and IL-22-producing T helper cells. The percentages of IL-22-producing CD4^+^CD45RO^+^ T cells in AH patients whose condition improved (**↓**GAHS ≥2) versus AH patients whose condition did not improve (**↓**GAHS <2) at days 0, 14 and 30 after diagnosis measured by flow cytometry. The frequency of these cells is elevated in the patient who experience disease improvement at baseline compared with the group without disease improvement (p<0.05). This elevation is persistent and augmented through the follow-up period (p<0.05). Values are presented as median (IQR). (GAHS: Glasgow alcoholic hepatitis score).

To examine the relationship between IL-22-producing CD4^+^CD45RO^+^ T cells and the course of AH, we divided the patients into two groups based on who experienced a short-term clinical improvement, which was defined as a decline in the GAHS ≥2 (n = 8), and who did not (n = 13). The patients whose condition improved exhibited approximately 2-fold higher frequencies of IL-22-producing CD4^+^CD45RO^+^ T cells on the day of diagnosis. This difference increased during the follow-up period, being most pronounced at 2 weeks with a 4-fold enrichment compared with the patients whose condition did not improve (p<0.05, Figure 4).

### Increased Plasma Concentration of IL-17A in AH Patients

In all of the study groups, we detected IL-17A-producing CD4^+^CD45RO^+^ T cells, but without difference in the frequencies of these cells among the groups. Nevertheless, the plasma concentration of IL-17A was 1.5-fold higher in the AH patients than in the healthy controls (*p*<0.01, Table 2); but a similar concentration was detected in the cirrhosis patients. The frequencies of IL-17A-producing CD4^+^CD45RO^+^ T cells and plasma IL-17A did not fluctuate during follow-up (Figure 3) and were not associated with changes in the GAHS (data not shown).

### IL-21 and IL-23 are not Elevated or Associated with the Course of Severe AH

We also measured IL-21-producing CD4^+^CD45RO^+^ T cells and the cytokines IL-21 and IL-23 in plasma, as they are involved in the induction and sustaining of the differentiation of Th17 cells. The frequency of IL-21-producing CD4^+^CD45RO^+^ T cells was not increased, and, similarly, the plasma concentrations of IL-21 and IL-23 did not differ among the groups. We did not observe any temporal differences in IL-21-producing CD4^+^CD45RO^+^ T cells or plasma IL-21 during follow-up or any association with GAHS improvement.

## Discussion

The results of the study did, indeed, provide new insights into the dynamics of the Th17-related cytokines during the course of severe AH: This study is the first to demonstrate high frequencies of IL-22-producing CD4^+^CD45RO^+^ T cells in patients with AH. In addition, our results may suggest a protective role of IL-22, because those patients whose condition improved exhibited consistently elevated frequencies of these cells. However, in disagreement with the second possibility we examined, our results seem to indicate that the proinflammatory IL-17A-producing CD4^+^CD45RO^+^ T cells, although present in AH, did not play a decisive part for the course of the disease.

Our study involved relatively few patients in whom a large number of measurements were conducted, and the results are not definitive or exhaustive. Nonetheless, some of the findings were quite consistent and quantitatively clear-cut. Our results, therefore, may be considered as an initial test of concept regarding the involvement of the Th17-related cytokines in the pathogenesis of AH, and we believe that our findings are of sufficient clarity to warrant further studies along the same line.

To date, there have been few studies on IL-22 in human liver disease. Elevations of T cells that produce IL-22 have been detected in patients with acute HBV at levels similar to those we observe in AH [24]. Additionally, IL-22-positive cells are found in HCV patients in both peripheral blood and liver tissue as well as in patients with hepatocellular carcinoma [25], [26]. Such point-studies can not be directly compared with our results but if confirmed would suggest that the IL-22 involvement may be a general feature of hepatic inflammation. In viral hepatitis, IL-22 facilitates hepatocyte survival according to observations in humans and IL-22 transgenic mice, and anti-apoptotic actions are proposed as an explanation for its upregulation in patients with hepatocellular carcinoma [16], [26], [27]. Regarding IL-17A, elevations have been detected in HBV, HCV, autoimmune hepatitis and primary biliary cirrhosis in which an IL-17A response is suggested to be disadvantageous [25], [28], [29], [30]. In human alcoholic liver disease, Lemmers et al. were the first to report the activation of the IL-17-pathway [18].

In this study, the AH diagnosis was established using a combination of standard clinical and biochemical criteria in accordance with several clinical trials [31], [32], [33]. All our AH patients were treated with pentoxyfilline according to the local clinical guidelines [34]. This drug induces a moderate decline in the production of TNF-α. However, the baseline samples were obtained prior to treatment and all AH patients received the same treatment; we find it unlikely, therefore, that the observed cell and cytokine differences between the groups resulted from the treatment.

The purpose of our work could be adequately addressed by means of cells from the blood, because the expression of IL-22 within the CD4^+^CD45RO^+^ sub-profile was so pronounced that the effects were clearly detectable in the periphery. Such blood-based data do seem to reflect events within the liver; a similar study on HCV patients found increased frequencies of IL-17A- and IL-22-producing T cells in the peripheral blood and even more so in the liver [25].

In this study, we report higher frequencies of circulating IL-22-producing CD4^+^CD45RO^+^ T cells in AH patients than in alcoholic cirrhosis patients and healthy controls. The reported functional effects of IL-22 are ambiguous and include context-dependent proinflammatory or tissue-preserving actions [10]. Nevertheless, the association between the improvement of the clinical disease course and the high frequency of IL-22-producing CD4^+^CD45RO^+^ T cells may be consistent with a beneficial role of these cells in AH. This would accord with investigations on liver inflammation in rodent models in which hepatic IL-22 mRNA, protein and receptor expression are increased, and where the blocking, knocking-out or neutralization of IL-22 accelerates the inflammatory injury [8], [16], [35].

The possible hepato-protective effects of IL-22 producing cells may not be exclusively related to alcohol-mediated inflammatory injury, because IL-22 has been suggested to play a protective role in both HBV, HCV and in models of acute liver failure [25], [26], [36]. The underlying mechanisms may be explained by *in vitro* studies of HepG2 hepatocellular carcinoma cells that demonstrated an IL-22-mediated induction of various anti-apoptotic and mitogenic proteins [35]. In mice, these IL-22 mediated effects resulted in amelioration of liver steatosis and fibrosis along with improved liver regeneration after tissue injury [37], [38], [39].

Despite the upregulation of IL-22-producing CD4^+^CD45RO^+^ T cells, we did not detect any change or difference in the plasma IL-22 levels in either of our analyses. This could result from sequestration of IL-22. In AH patients, hepatic IL-22 membrane-associated receptors are increased, and furthermore a soluble decoy receptor, IL-22-binding-protein, exists, which holds a higher binding affinity than the functional membrane receptor [17], [40]. LPS, usually highly elevated in AH, is known to increase the expression of this decoy receptor [41].

In regards to the potent proinflammatory Th17 cytokine IL-17A, we confirmed the presence of IL-17A in AH as shown by Lemmers et al. [18]. We found a lower plasma level of IL-17A as also others have done, and the difference may depend on the ELISA assays [22], [25]. We observed no change over time in the cell frequencies or the plasma concentrations of IL-17A. This may argue against a dominant role of IL-17A for the disease course of AH, in agreement with our results on the cytokines, IL-21 and IL-23, involved in Th17 cell differentiation. For IL-23, the majority of the patients had undetectable levels, and levels of plasma IL-21 and IL-21-producing CD4^+^CD45RO^+^ T cells were not increased and did not correlate with disease markers in AH.

The current therapeutic strategy of prednisolone or pentoxifylline is double-edged, because the beneficial anti-inflammatory actions of these drugs are accompanied by increased susceptibility to infections and inhibited liver regeneration [32], [42]. In accordance with the described biological roles of IL-22, the cytokine has been suggested as an add-on treatment option that could compensate for the adverse effects of the clinical immunomodulators [43]. Our findings, although explorative of nature, may lend support to such an approach, but first of all larger studies involving hard clinical outcomes such as mortality and cirrhosis are needed.

In conclusion, we found increased frequencies of IL-22-producing T helper cells in the patients with AH and most so in the patients with clinical disease improvement. We suggest this to support the emerging concept of IL-22 as a hepato-preserving cytokine with a role in the course of AH.
